# The Correlation of Clinicopathological Findings and Neutrophil-to- Lymphocyte and Platelet-to-Lymphocyte Ratios in Papillary Thyroid Carcinoma

**DOI:** 10.4274/mirt.galenos.2018.60490

**Published:** 2019-03-19

**Authors:** Yeşim Ceylan, Kamil Kumanlıoğlu, Aylin Oral, Yeşim Ertan, Zehra Özcan

**Affiliations:** 1 Adıyaman Faculty of Medicine Training and Research Hospital, Department of Nuclear Medicine, Adıyaman, Turkey; 2 Ege University Faculty of Medicine, Department of Nuclear Medicine, İzmir, Turkey; 3 Ege University Faculty of Medicine, Department of Pathology, İzmir, Turkey

**Keywords:** Neutrophil-to-lymphocyte ratio, platelet-to-lymphocyte ratio, papillary thyroid carcinoma

## Abstract

**Objectives:**

Inflammatory markers such as neutrophil-to-lymphocyte ratio (NLR) and platelet-to-lymphocyte ratio (PLR) have been recently introduced as potential biomarkers for tumor pathogenesis, development and prognosis in solid tumors. Our aim was to assess the correlation of clinicopathological features and NLR and PLR in patients with papillary thyroid carcinoma (PTC).

**Methods:**

A total of 201 papillary thyroid carcinoma patients were divided into groups with a cut-off preoperative median NLR and PLR value of 1,92 and 123.9, respectively. The correlation of NLR and PLR and clinicopathological features including age, tumor size, extra-thyroidal extension, thyroid capsule invasion, surgical margin positivity, multifocality, bilaterality of the patients were analyzed.

**Results:**

The mean NLR and PLR were 2.11±0.94, 129.69±42.81, respectively. Larger tumor size and higher positivity of extra-thyroidal spread were correlated with higher NLR values. No significant relationship was found between NLR and age, presence of thyroid capsule invasion, surgical margin positivity, multifocality, bilaterality, and lymph node metastasis. Also no significant association was observed between the clinicopathological features and PLR.

**Conclusion:**

High NLR was found to correlate with tumor size and extra-thyroidal extension. NLR may be used as a marker to determine the clinical behavior of disease in patients with papillary thyroid carcinoma (PTC).

## Abstract (tr)

**Amaç:**

Son yıllarda nötrofil/lenfosit oranı (NLO) ve trombosit/lenfosit oranı (TLO) gibi enflamatuvar belirteçlerin solid tümör patogenezinde, gelişmesinde ve prognozunda etkili olduğuna dair çalışmalar mevcuttur. Bu çalışmada tiroid papiller kanserinin (PTK) kliniko-patolojik özellikleri ile NLO ve TLO arasındaki ilişkinin retrospektif olarak incelenmesi amaçlanmıştır.

**Yöntem:**

Çalışmaya dahil edilen 201 hasta preoperatif medyan NLO (grup 1 <1,92 ve grup 2 ≥1,92) ve medyan TLO (grup 1 <123,9 ve grup 2 ≥123,9) değerlerine göre gruplara ayrıldı. NLO ve TLO ile hastaların yaş, tümör boyutu, ekstra-tiroidal yayılım, tiroid kapsül invazyonu, cerrahi sınır pozitifliği, multifokalite, bilateralite gibi kliniko-patolojik özellikleri arasındaki ilişki değerlendirildi.

**Bulgular:**

Ortalama NLR ve PLR sırasıyla 2,11±0,94, 129,69±42,81 idi. Verilerin istatiksel analizi preoperatif yüksek NLO ile tümör boyutu (p=0,002) ve ekstra-tiroidal yayılım (p=0,028) arasında anlamlı ilişki bulunduğunu gösterdi. Yaş, tiroid kapsül invazyonu, cerrahi sınır pozitifliği, multifokalite, bilateralite ile NLO arasında ise ilişki saptanmadı (p>0,05). TLO ile kliniko-patolojik özellikler arasında anlamlı istatistiksel ilişki gösterilemedi (p>0,05).

**Sonuç:**

Çalışmamız PTK de NLO ile tümör boyutu ve ekstra-tiroidal yayılım arasında istatistiksel olarak anlamlı ilişki bulunduğunu göstermektedir. NLO’nun diğer bazı solid tümörlerde olduğu gibi PTK olgularında hastalığın klinik davranışını belirlemek için yararlı bir belirteç olarak kullanılabileceği düşünülmektedir.

## Introduction

It has been demonstrated that inflammation might play an important role in cancer development and progression (1). The interaction between cancer and inflammation is assumed to be complicated and based on different physiological processes such as miscellaneous inflammatory cells, mediators and signaling pathways in cancer tissue (2). It has been indicated that cancer-related inflammatory response leads to proliferation and survival of tumor cells, angiogenesis and finally to cancer progression by affecting tumor microenvironment in numerous tumors (3). The increase of pro-inflammatory cytokine is regarded to be indicative of disease prognosis and patient response to the tumor. Thus, systemic inflammatory markers including C-reactive protein (CRP), albumin concentration, neutrophil-to-lymphocyte ratio (NLR), platelet-to-lymphocyte ratio (PLR) may have potential roles as prognostic biomarkers (4).

NLR, which is simply measured by a routine peripheral blood test, has been widely used as an indicator of general immunoreactivity. It has been studied in various tumors and found to be useful as a prognostic indicator, estimating overall and recurrence free survival in some solid tumors such as esophagus, stomach, pancreas, colon, ovary, kidney, lung and prostate cancers (5,6,7). However, studies examining the role of NLR in thyroid cancer with an increasing frequency worldwide are limited. In the current study, we aimed to evaluate the correlation of clinicopathological features and inflammatory indicators in papillary thyroid cancer.

## Materials and Methods

### Patients

The study group included papillary thyroid carcinoma patients referred to Department of Nuclear Medicine between January 2015 and December 2016. Those patients with confirmed diagnosis of thyroid papillary carcinoma greater than 1 cm on a detailed histopathological examination and total blood count analysis just prior to thyroid surgery (within a 2 days interval) were selected. Patients with co-existing hematologic diseases, additional tumors, acute myocardial infarction or coronary revascularization in the last 6 months, acute infectious diseases, chronic drug (steroids etc.) use that could affect blood analysis, presence of lymphocytic infiltration suggesting thyroiditis on histopathology and abnormal white blood cells (WBC) measurements were excluded from the study. The medical records of all patients were examined and those without symptoms of acute infections and normal blood cells were included. The final study population included a total of 201 patients. Demographic characteristics of the patients (age, gender), clinical records including histopathologic findings, and pre-operative complete blood count results were obtained. All surgical specimens were examined in detail for certain pathologic features including tumor size, presence of thyroid capsule invasion, extra-thyroidal extension, surgical margin positivity, bilateral involvement, presence of multifocal tumor and lymph node metastasis. We used complementary data achieved by ultrasound and post ablation whole body iodine scan to assess lymph node involvement as neck dissection was not routinely performed to all patients. Complete blood count analyzes; hemoglobin level, WBC, neutrophil and lymphocyte counts were obtained by using a Dyn Ruby Cell (ABBOTT, USA) hematology analyzer. The NLR was calculated by dividing the absolute neutrophil count by the absolute lymphocyte count; similarly, the PLR was calculated by dividing the absolute platelet count by the absolute lymphocyte count. We formed 2 cohort groups according to the values above and below the median value of NLR and PLR. These groups were compared in terms of the aforementioned clinicopathologic characteristics.

The study was approved by the Ege University of Local Ethics Committee (protocol number: 17-12.1/33).

### Statistical Analysis

Statistical Package for Social Sciences version 15.0 (SPSS Inc., Chicago, IL, USA) was used for statistical analysis. Kolmogorov-Smirnov test was used to determine if sample data is normally distributed. The Mann-Whitney U test was then used to compare the continuous variables which did not show normal distribution. The correlation between the nominal variables was compared with the chi-square test. P value less than 0.05 was considered statistically significant.

## Results

An overview of patient characteristics is shown in Table 1. The mean age of the study population was 47.1±14.3 years, and the female/male ratio was 155/46. Two of the patients had distant metastatic involvement (lung) and 57 had cervical lymph node metastases.

The mean NLR and PLR were 2.11±0.94 and 129.69±42.81, respectively. The patients were divided into two groups according to the median NLR as those below (group 1) and above (group 2) 1.92. When clinic-pathologic features were compared by using chi-square and Mann-Whitney U tests, larger tumor size (group 1: 2.24±1.14 cm; group 2: 2.79±1.49 cm, p=0.002), and higher positivity of extra-thyroidal spread (group 1: 3 patients, group 2: 11 patients, p=0.028) were found to be statistically related with the higher values of NLR in group 2. Statistical analyses did not reveal a significant correlation between NLR and age (<45 years, ≥45 years), presence of thyroid capsule invasion, surgical border positivity, multifocality, bilaterality, lymph node metastasis (Table 2). When the cohort was also divided into two groups according to median PLR (PLR <123,9 and PLR ≥123,9), no statistically significant correlation was detected with clinic-pathologic features (Table 3).

## Discussion

It has been widely recognized that inflammation and cancer are closely related to each other as inflammation has both cancer-inhibiting and neoplasia modelling properties (8,9,10). The inflammatory effect on tumor pathogenesis, which was first described by Rudolf Virchow, has been recognized as an important concept also for the development and proliferation of the tumor by reducing response to anticancer agents (11,12). In recent studies, there is growing evidence on the effect of inflammation on cancer pathogenesis, progression and response to treatment (2,12). Inflammation, cytokines and chemokines induce tumor proliferation, angiogenesis and metastasis by CRP and neutrophil induction. In addition, it is considered to play an important role in the development and proliferation of the tumor by reducing the response to anticancer agents (2). The physiologic response of leukocytes to stress results in an increase in the number of neutrophils and a decrease in the number of lymphocytes (13). The inflammatory cytokines, leukocytes and phagocytic mediators that cause neutrophil release, lead to DNA damage. It inhibits apoptosis and induces tumor angiogenesis resulting in tumor growth, progression, and metastasis. On the other hand, lymphocytes also play a major role in the prevention of tumor growth and immunity (10).

Recently some studies pointed out that elevated blood NLR that can be easily calculated from blood tests might be used to predict some aggressive features in a variety of cancers (5,6,7). In the current study, we aimed to examine the value of NLR and PLR in papillary thyroid cancers in which the current relevant literature is quite limited (14,15,16). 

In the current study patients with papillary thyroid carcinoma, we have noted a statistically significant association between high preoperative NLR value and size and extra-thyroidal extension of the tumor. This observation was in agreement with the study of Manatakis et al. (8) indicating that the high levels of NLR was associated with extra-thyroidal invasion. Also, Liu et al. (16) showed the correlation of high preoperative NLR values with increased tumor size and recurrence risk in differentiated thyroid cancers. Several additional studies have also supported the correlation of tumor size and increasing NLR (14,15,16). However, in our study, pathologic findings other than tumor size and extra-thyroidal extension did not appear to be inter-related. Moreover, no correlation was found between PLR and clinic-pathologic features of thyroid tumors. This inconsistent observation might be related to several factors related to the study population and methodology. Moreover, as inflammation is a slow process and most of the study patients herein represent early stage of the disease, no significant correlation between inflammation and NLR was noted. In the study of Manatakis et al. (8), the study group (205 patients) included those cases with tumors smaller than 1 cm and those with co-existing thyroiditis. Actually this is divergent from our cases as we have excluded smaller tumors and those with thyroiditis. In the study of Gong et al. (14), the median NLR used as a cut-off value was 2.0 that is similar to our study. They have found a positive correlation between high NLR and lymph node metastasis, multifocality and tumor size. However it should be noted that these NLR values obtained in the studies focusing on thyroid carcinoma are lower than those in previous studies focusing on solid tumors. As an example, Templeton et al. (17) found the median NLR value as 4 in a meta-analysis with solid tumors. Moreover, it should also be considered that there has been no clear validation of the cut-off values used in the literature (14). Regarding the correlation between disease extension and NLR, Manatakis et al. (8) and Gong et al. (14) found an association between the presence of lymph node metastases, which is not supported in our series. As stated above, this might be linked to the differences in the study population and the number of patients with lymph node involvement in their series which is obviously smaller than ours. On contrary to this, Kim et al. (15) have indicated lack of evidence for the association between NLR and the clinicopathological findings of the tumor based on 1066 female patients. However, they have found a significant correlation between high pre-operative PLR and lymph node metastasis. In the current study, while a significant correlation between NLR and tumor size and extra-thyroidal extension was noted, an association with PLR was not detected. In most of the previous studies, both NLR and PLR were found to be valuable in several solid tumors (18,19,20,21). Costantini et al. (22) suggested that production of bone marrow-stimulating cytokines as a result of inflammatory response to malignancy may play an important role in the regulation of platelet counts in neoplasms (23). Platelets can give rise to angiogenesis and extra-vasation of tumor cells by releasing vascular endothelial growth factor (VEGF) (24). VEGF and various growth factors have been suggested to induce angiogenesis and vascularization resulting in the increase of tumor growth rates (25). Some proinflammatory cytokines, such as IL-1 and IL-6, also cause megakaryocyte proliferation resulting in thrombocytosis (24,25). 

Several clinical studies showed that high PLR correlates with worse clinicopathological features in patients with HCC (26,27). Deng et al. (28) performed a literature search in PubMed, Web of Science and Embase. This meta-analysis included 13 studies involving 4.621 patients. The result indicated that the elevated PLR level was associated with lymph node metastasis, higher tumor stage, deeper tumor invasion and longer tumor length, indicating that the level of PLR is important for predicting clinicopathological features. Most of the studies have been performed in esophagus, ovary, breast, prostate, stomach, colorectal and hepatocellular carcinomas with limited studies focusing on thyroid cancer (29,30). Previously Kim et al. (15) documented elevated PLR in association with increased risk of lateral lymph node involvement. However, when combining NLR and PLR, they were not able to support the correlation of these markers with prognostic factors in papillary thyroid carcinoma. A high preoperative PLR is associated with poor prognosis in operable colorectal and pancreatic cancers (19), and a high preoperative NLR is poor prognostic marker in some cancers, including gastric, pancreatic, colorectal, cholangiocarcinoma, lung and ovarian cancers (6). But only a few studies have evaluated the significance of the NLR and also PLR in thyroid cancer. Measurement of the PLR and NLR were cost-effective, safe, and readily available so we evaluated the association between preoperative NLR and PLR and the clinicopathological characteristics of patients with PTC. Unfortunately; it should be considered that this study has some limitations related to the limited number of patients and retrospective study design. Also, NLR and PLR values are not specific for inflammation process and may be affected by many factors. Moreover, lack of standard cut-off values for NLR and PLR also appear to be important to validate these observations. Another limitation is that patients who had PTC below 1 cm have not been investigated in this study although tumors below 1 cm may have metastasis or extra-thyroidal invasion. Further studies including thyroid papillary microcarcinomas may provide future guidance.

## Conclusion

In the current analysis, we identified a statistically significant correlation between NLR and tumor size and extra-thyroidal extension. However, no evidence of correlation with these features and PLR was observed. The current results indicate NLR, which is a quite simple and inexpensive test, as a potential marker to determine clinical behavior in papillary thyroid carcinoma patients.

## Figures and Tables

**Table 1:**
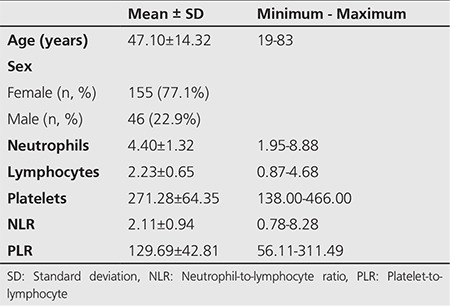
Demographic characteristics and hematological data of papillary thyroid carcinoma patients

**Table 2:**
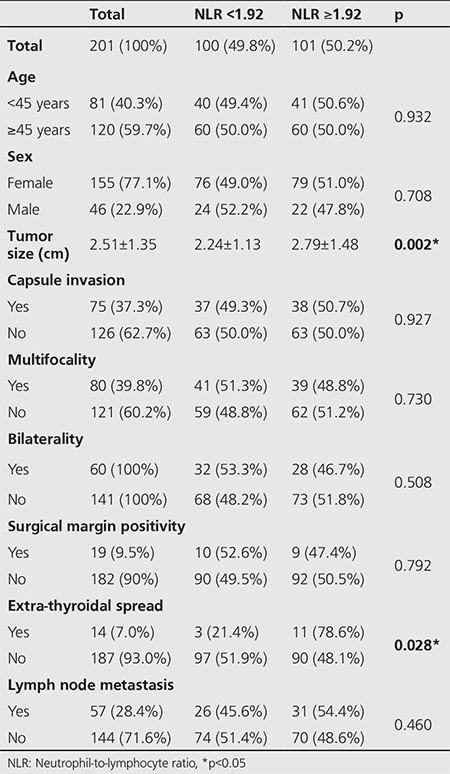
Association of preoperative neutrophil-tolymphocyte ratio with clinicopathological characteristics of papillary thyroid carcinoma

**Table 3:**
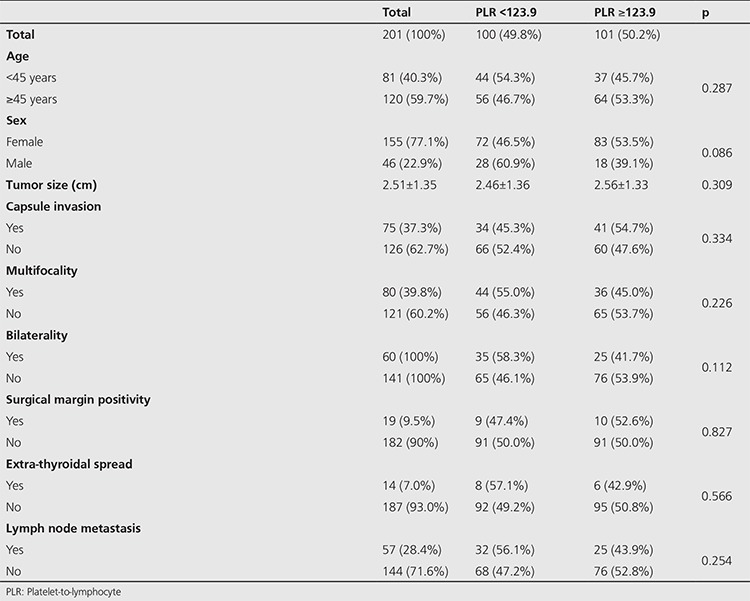
Association of preoperative platelet-to-lymphocyte with clinicopathological characteristics of papillary thyroid carcinoma
